# Silicone Cervical Lymphadenopathy: A Rare Complication After Breast Augmentation

**DOI:** 10.7759/cureus.50453

**Published:** 2023-12-13

**Authors:** Caroline-Theodora Avgeri, Giorgos Sideris, Ioannis Margaris, Laith Tapponi

**Affiliations:** 1 ENT Department, Royal Free London NHS Foundation Trust, London, GBR; 2 2nd ENT Department, Attikon University Hospital, National and Kapodistrian University of Athens, Athens, GRC; 3 4th Department of Surgery, National and Kapodistrian University of Athens, Athens, GRC

**Keywords:** breast augmentation, silicone, breast implant, granuloma, lymphadenopathy

## Abstract

A 66-year-old female patient, who had undergone breast augmentation 10 years ago, presented with unilateral neck pain, dysphagia, and hoarseness. Subsequent imaging revealed right-sided supraclavicular and axillary lymphadenopathy and a breast implant rupture on the same side. A lymph node core biopsy under sonographic guidance revealed silicone lymphadenopathy. Implant extirpation was offered to the patient. Cervical lymphadenopathy warrants an initial workup to exclude sinister chronic inflammatory or malignant conditions. Nonetheless, in cases of diagnostic uncertainty and a history of breast augmentation, the otolaryngologist should be cognizant of distal silicone lymphadenopathy. This condition is associated with silicone leakage and lymphatic dissemination of silicone particles. Even though silicone-related granuloma formation is a rare entity, its incidence is slowly rising as the population that has undergone breast augmentation grows older.

## Introduction

The presence of unilateral cervical lymphadenopathy should always raise suspicion of cancer, especially when accompanied by alarming symptoms such as dysphagia or hoarseness. In such a clinical scenario, a detailed physical examination and patient workup are warranted [1]. The differential diagnosis includes inflammatory processes of the head and neck, primary hematologic neoplasms, metastatic diseases, connective tissue disorders, and other infrequent etiologies. In a large retrospective analysis of 262 patients who underwent diagnostic cervical lymphadenectomy, hematologic neoplasia was present in 35% of the patients, while metastatic disease was identified in 25% of them [2]. We report an interesting case of cervical lymphadenopathy associated with silicone breast augmentation and provide a brief review of the literature. Even though silicone granuloma is a well-described complication of ruptured breast implants, it most commonly manifests as lymphadenopathy confined to the axillary nodal basin [3]. Only a few similar cases of silicone neck lymphadenopathy have been reported so far.

## Case presentation

A 66-year-old female patient presented to the outpatient ear, nose, and throat (ENT) clinic of the Royal Free London NHS Foundation Trust in March 2023 with a two-month history of right-sided neck pain, intermittent hoarseness, dysphagia, constant need to clear her throat, and ear pain while eating and drinking. Her past medical history included gastroesophageal reflux disease, fibromyalgia, and bilateral breast augmentation for cosmetic reasons 10 years ago. The patient was not a smoker and denied any history of unintentional weight loss, fevers, or night sweats. The physical examination was generally uneventful; no lumps were observed, whereas the only finding on endoscopy was the presence of laryngeal oedema indicative of reflux, which was considered the main cause of her symptoms. Dietary lifestyle modifications were recommended, and an anti-reflux medication was prescribed, along with an MRI of the neck.

**Figure 1:**
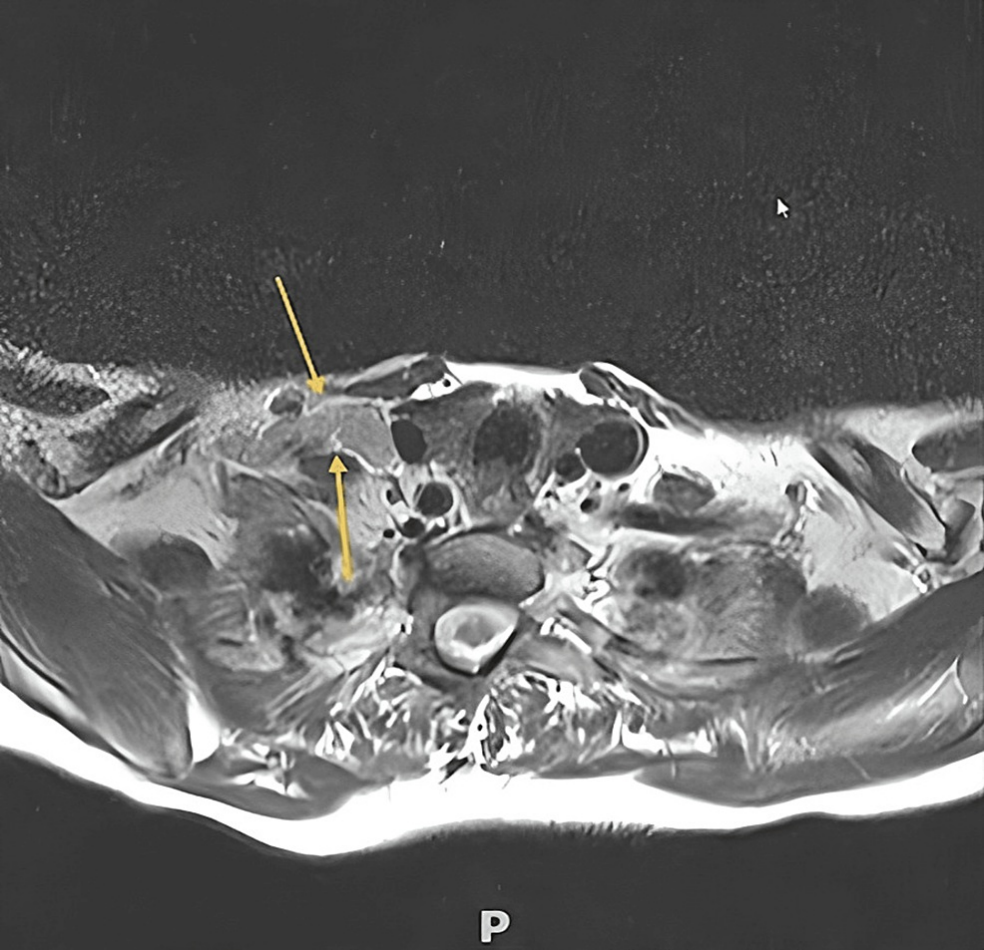
Patient’s neck MRI depicting the pathologically enhanced cervical lymph nodes (yellow arrows) MRI: magnetic resonance imaging

Her general practitioner also referred the patient to a gastroenterologist around the same time. Subsequently, she underwent oesophagogastroscopy (OGD), which indicated the presence of gastritis and fundic gland polyps. Histopathology revealed reactive gastritis with minimal mucosal inflammation of the stomach and mild inflammatory/reflux-related changes of the oesophagus. The MRI scan showed evidence of indeterminate right-sided lower cervical lymphadenopathy that could be either of infective or malignant origin. More specifically, there were pathologically enhanced but non-necrotic lymph nodes in the right IV and Vb neck levels, measuring up to 13 mm in short axis diameter (Figure 1).

An ultrasound scan of the area demonstrated well-defined hyperechoic cervical lymph nodes with posterior acoustic shadowing. A targeted core biopsy of a posterior triangle lymph node revealed the presence of granulomatous inflammation, comprising multinucleated giant cells intermingled with vacuolated cells, many of which were likely histiocytes. No refractive material was identified by polarized light microscopy. Several giant cells featured prominent asteroid bodies. Necrotising granulomatous inflammation of microbial etiology was not evident. The conclusion was that granulomatous inflammation was likely secondary to an exogenous material.

**Figure 2:**
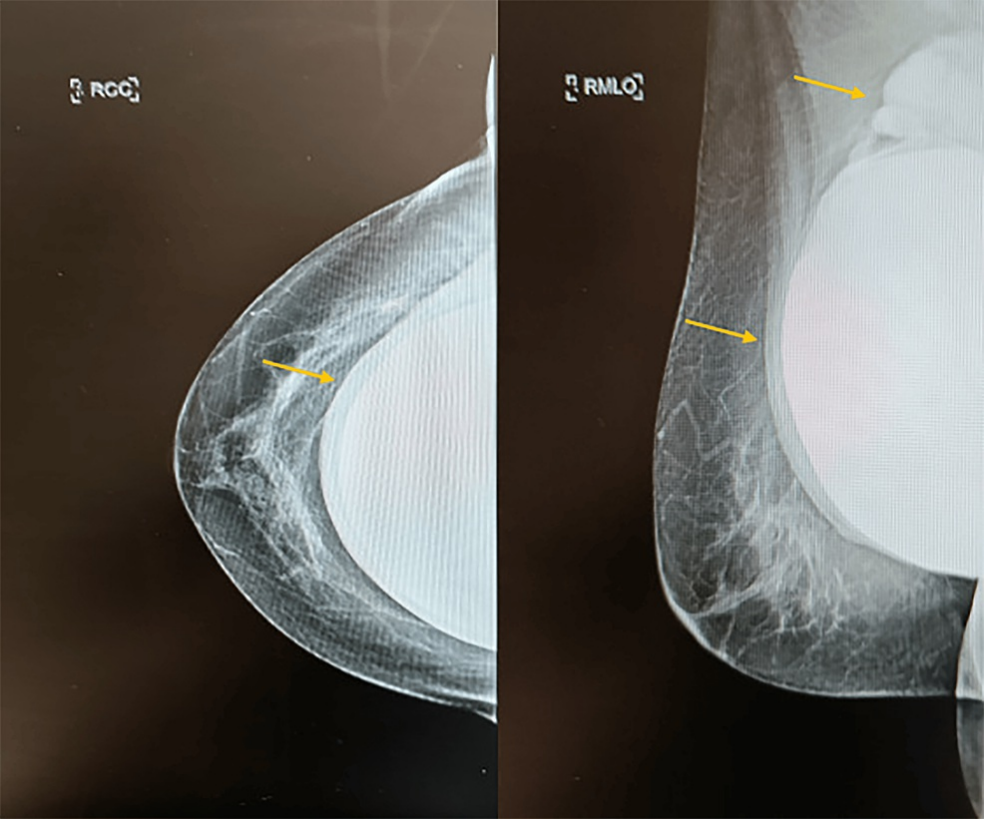
Patient’s right craniocaudal (RCC) and mediolateral oblique (RMLO) mammographic views The images depict the right implant capsule thickening and a lobulated density superior to the implant extending towards the axilla (yellow arrows)

The case was further discussed at the head and neck multidisciplinary team (MDT) meeting, and a referral to the breast clinic was agreed upon. Reviewing the patient’s last mammographic study, a lobulated density was seen superior to the right implant and extending towards the lower axilla. Furthermore, the implant capsule appeared thickened on the right side (Figure 2).

The overlying breast parenchyma demonstrated a fatty glandular pattern without any focal or suspicious features. Appearances were suggestive of right implant rupture, and further assessment was carried out with an ultrasound, which confirmed the diagnosis and also revealed the presence of axillary lymphadenopathy. Hence, the patient was diagnosed with silicone-related cervical lymphadenopathy. She was offered conservative symptomatic treatment with warm compresses, to alleviate her neck pain, and breast implant extirpation. Since then, she has been followed up at the outpatient clinics. During her last visit, she remained well and symptom-free.

## Discussion

Isolated cervical lymphadenopathy is a common reason for ENT referral and assessment. Although most of the cases are attributed to benign conditions, physical examination and workup are warranted to rule out the possibility of a primary or secondary malignancy or an insidious chronic inflammatory condition [1]. Given that cervical lymph nodes (including the supraclavicular nodes) receive drainage from the head, neck, breast, and upper abdomen, they can harbor malignancy originating from any of the above-mentioned areas [4].

Our patient presented with worrying symptoms of hoarseness, dysphagia, and unilateral neck pain. She underwent further evaluation and was eventually found to have cervical lymphadenopathy associated with a silicone implant rupture on the same side. Lipogranulomatous inflammation of the axillary lymph nodes after breast augmentation is a well-known late complication, usually occurring six to eight years following implantation [3]. Similar problems have been reported by ophthalmologists and orthopaedists, who often encounter cases of granuloma formation after vitrectomy, silicone oil retinal tamponade or injection of silicone in joints, and silicone joint prosthesis operations [5-6]. However, only a few reports exist on cervical lymphadenopathy associated with breast implants, and these mainly involve single case reports or small case series [7-15] (Table 1).

**Table 1:** Similar reports of cervical silicone-related lymphadenopathy in the literature

Study	Number of patients	Background	Manifestations	Diagnosis	Treatment and outcomes
Khakbaz et al. [7]	One case	15 years after saline-based implantation (initially silicone implants, subsequently changed – implant ruptures)	Right-sided cervical and axillary lymphadenopathy	Excisional biopsy	-
Rajgor et al. [8]	One case	10 years after saline-based implantation	Isolated cervical (levels IV, Vb) lymphadenopathy bilaterally; attributed to ‘’gel bleed’’	Core biopsy	No further treatment
Borghol et al. [9]	One case	10 years after silicone Poly Implant Prosthése (PIP) implantation	Isolated right-sided supraclavicular lymphadenopathy; implant rupture	Fine needle aspiration, core biopsy, excisional biopsy	Lymph node excision, implant removal
Gilbert and Thiruchelvam [10]	One case	10 years after silicone implantation (left implant replaced two years before presentation, due to leakage)	Left-sided supraclavicular and axillary lymphadenopathy	Core biopsy	No further treatment
Mistry et al. [11]	One case	5 years after silicone Poly Implant Prosthése (PIP) implantation	Bilateral cervical, axillary, and internal mammary lymphadenopathy; thoracic outlet syndrome; bilateral implant rupture	Excisional biopsy	Corticosteroids, cervical and axillary lymph node clearance, implant explantation, and capsulectomy
Garcia Callejo et al. [12]	Twelve cases	8-70 months after silicone implantation	Cervical lymphadenopathy, with additional axillary/mediastinal/ internal mammary lymphadenopathy in 8 patients (67%); silicone leakage confirmed in 7 cases (58%)	Fine needle aspiration	Conservative in 7 cases (58%), cervical lymph node excision in 5 cases (42%); removal of implants in all cases; in 2/7 cases (29%), increase in size with conservative treatment; in 2/5 cases (40%), recurrence after cervical lymph node excision
Kolios et al. [13]	One case	5 years after silicone Poly Implant Prosthése (PIP) implantation	Right-sided cervical and axillary lymphadenopathy; right implant rupture	Excisional biopsy	Bilateral implant removal and capsulectomy, axillary lymph node dissection
Omakobia et al. [14]	One case	13 years after silicone implantation	Isolated left supraclavicular (level IV) lymphadenopathy; bilateral implant rupture	Fine needle aspiration, excisional biopsy	No further treatment, referral to plastic surgeon
Shipchandler et al. [15]	One case	6 years after saline-based implantation (initially silicone implants, subsequently changed due to painful capsular contraction)	Right-sided supraclavicular and bilateral axillary lymphadenopathy	Fine needle aspiration, excisional biopsy	Cervical lymph node excision

Overall, 20 similar cases have been reported in the literature so far. The duration from breast implantation to silicone cervical lymphadenopathy diagnosis ranged between eight months and 15 years. Pathological lymph nodes were usually located in the right supraclavicular area, although there have been reports of bilateral cervical lymphadenopathy [8,11]. Coexistent axillary lymphadenopathy was usually evident, corresponding to the route of breast lymphatic drainage. However, there have been cases of isolated cervical lymphadenopathy as well [8-9,12,14]. In general, both silicone implants and saline-based implants, the latter containing only an outer silicone shell, have been implicated. It is noteworthy that the use of silicone Poly Implant Prosthése (PIP) breast implants was suspended in 2010, after concluding that the silicone gel used by PIP was not medical grade. They had been associated with significantly higher rates of implant rupture and silicone lymphadenopathy [9,11,13,16]. In terms of histological diagnosis of silicone cervical lymphadenopathy, lymph node fine needle aspiration, core biopsy, and excisional biopsy, alone or in combination, have been used. In the majority of cases, no further treatment or conservative treatment was offered to the patients. 

Implant rupture is reported in approximately 1% of patients with silicone implants [9]. The ensuing erosion can result in either intracapsular confined leakage or extravasation of the material into the breast tissue. Even a microscopic silicone leakage from the outer shell (termed ‘’gel bleed’’) has been described, which is particularly relevant to cases of saline-based implants, as already mentioned. Silicone particles can then migrate to regional lymph nodes via macrophages of the reticuloendothelial system, resulting in swelling, fibrosis, and a foreign body granulomatous reaction. The classic histopathology report describes the presence of giant cells, asteroid bodies, lymphoid infiltration, and clear cytoplasmic silicone vacuoles [17]. In terms of diagnosis, fine needle aspiration or core biopsy of an enlarged lymph node is considered the initial study of choice and may be sufficient. When necessary, an open excisional biopsy can be performed.

No published guidelines are currently available for the treatment of silicone lymphadenopathy. Conservative symptomatic treatment is generally recommended, consisting mainly of warm compresses to relieve the pain, which may obviate the need for any systematic therapy. Lymph node excision or clearance may be indicated in cases of persistent symptoms or diagnostic uncertainty, but the condition may recur even after the surgical removal of the diseased nodes [11-12]. Alternative strategies have been proposed for the management of silicone lymph node granulomas, albeit with minimal evidence to support their wide adoption. They include pharmacologic agents such as corticosteroids, minocycline, cyclosporine, isotretinoin, imiquimod, and the use of CO_2_ lasers. The latter can vaporise granulomas by overcoming the thermostability of the material, but it carries the risk of lymph node necrosis [18]. Breast implant extirpation and capsulectomy may also be offered to the patient to prevent future recurrences.

Silicone is a permanent, thermostable, non-carcinogenic filler with a limited risk of bacterial growth, stable viscoelasticity, and minimal adhesivity to surrounding tissues. Despite the apparently beneficial material profile, its properties may prove disadvantageous in cases of implant rupture and subsequent dissemination. Complications, such as granuloma formation, cellulitis, skin ulcers, necrosis, and even hepatitis or pneumonitis in immunocompromised patients, have been reported. Lastly, one should not disregard the infrequent, though established, association between implants and anaplastic large cell lymphoma (ALCL). The latter commonly presents as a unilateral breast effusion or mass. Associated axillary lymphadenopathy may be present in a small proportion of patients; yet, less frequently, supraclavicular lymphadenopathy may be evident, which is classified as a clinical-stage II lymphoma based on the traditional Ann Arbor staging system [19-20].

## Conclusions

Breast mammoplasty with the use of implants is considered an attractive, popular, and safe plastic surgery procedure. However, silicone leakage can result in lymphatic dissemination of silicone particles, most commonly towards the axillary nodal basin. We reported a rare case of a patient who presented with symptomatic cervical lymphadenopathy, due to silicone granuloma formation. Her imaging studies revealed the presence of ipsilateral breast implant rupture and the diagnosis of silicone cervical lymphadenopathy was confirmed based on a targeted lymph node core biopsy. Cervical lymphadenopathy warrants an initial evaluation to exclude severe chronic inflammatory or malignant conditions. Nonetheless, in cases of diagnostic uncertainty and a history of breast augmentation, the otolaryngologist should be cognizant of distal silicone lymphadenopathy.
